# TLR-4 Signaling vs. Immune Checkpoints, miRNAs Molecules, Cancer Stem Cells, and Wingless-Signaling Interplay in Glioblastoma Multiforme—Future Perspectives

**DOI:** 10.3390/ijms21093114

**Published:** 2020-04-28

**Authors:** Jakub Litak, Cezary Grochowski, Joanna Litak, Ida Osuchowska, Krzysztof Gosik, Elżbieta Radzikowska, Piotr Kamieniak, Jacek Rolinski

**Affiliations:** 1 Department of Neurosurgery and Pediatric Neurosurgery, Medical University of Lublin, 20-954 Lublin, Poland; 2 Department of Immunology, Medical University of Lublin, 20-093 Lublin, Poland; 3 Department of Anatomy, Medical University of Lublin, 20-090 Lublin, Poland; 4 Laboratory of Virtual Man, Department of Anatomy, Medical University of Lublin, 20-090 Lublin, Poland; 5 St. John‘s Cancer Center in Lublin, 20-090 Lublin, Poland; 6 MSWiA Hospital, 02-507 Warsaw, Poland

**Keywords:** glioblastoma multiforme, TLR, TLR-4, toll-like receptor, glioma, high-grade glioma

## Abstract

Toll-like-receptor (TLR) family members were detected in the central nervous system (CNS). TLR occurrence was noticed and widely described in glioblastomamultiforme (GBM) cells. After ligand attachment, TLR-4 reorients domains and dimerizes, activates an intracellular cascade, and promotes further cytoplasmatic signaling. There is evidence pointing at a strong relation between TLR-4 signaling and micro ribonucleic acid (miRNA) expression. The TLR-4/miRNA interplay changes typical signaling and encourages them to be a target for modern immunotherapy. TLR-4 agonists initiate signaling and promote programmed death ligand-1 (PD-1L) expression. Most of those molecules are intensively expressed in the GBM microenvironment, resulting in the autocrine induction of regional immunosuppression. Another potential target for immunotreatment is connected with limited TLR-4 signaling that promotes Wnt/DKK-3/claudine-5 signaling, resulting in a limitation of GBM invasiveness. Interestingly, TLR-4 expression results in bordering proliferative trends in cancer stem cells (CSC) and GBM. All of these potential targets could bring new hope for patients suffering from this incurable disease. Clinical trials concerning TLR-4 signaling inhibition/promotion in many cancers are recruiting patients. There is still a lot to do in the field of GBM immunotherapy.

## 1. Introduction

Various toll-like-receptor (TLR) family members are detected in the central nervous system (CNS). They are mainly expressed in neurons and glial structures, where they play a role in recognizing unfamiliar molecules, some postapoptotic antigens consequently control repair processes and modulate inflammatory action [1].

Furthermore, TLR occurrence was noticed and widely described in glioblastoma-multiforme (GBM) mature cell fractions [2]. Studies revealed a strong expression of these receptors in cohorts of neural non-pro-oncogenic stem cells [3,4]. GBM, as the most popular and expansive glioma form, with a mean survival of 14.6 months, remains a challenge for modern therapy [5]. The characteristics of TLRs make them a promising target for GBM immunotreatment. Activated receptors stimulate the response of the immune system and control the course of many diseases, including cancers [6,7,8,9]. The necrotic ratio during glioblastoma invasion remains high. Products of cell breakdown intensively interact with the transmembrane architecture of TLRs and promote differentiation and inflammatory signaling [10]. In brain tissue, TLR-4 is detected in two main types of cells: microglial (macrophages) and macroglial cells (oligodendrocytes and astrocytes). Microglia do not intensively express TLR-4 on the surface (less than 15%), and receptors are easier to detect intracellularly. Macroglial cells superficially express TLR-4. Oligodendrocytes and astrocytes do not intracellularly express TLR-4 at all. This difference in expression could be explained by the different phagocytic functions of micro- and macroglial cells (Table 1) [11,12].

The TLR family is a group of ten receptors (TLR-1–TLR-10) characterized by the detection of a particular pattern of micro-organisms (pathogen-associated molecular patterns (PAMPs)), which are invariable for most pathogens and not present in mammalian organisms. TLR receptors are also sensitive to particles secreted during necrosis and cell death called danger-associated molecular patterns (DAMPs) [20,21,22,23]. Typically, TLRs are grouped into two major categories, endosomal (TLR-9, TLR-8, TLR-7, and TLR-3) and cell-surface-acting (TLR-10, TLR-6, TLR-5, TLR-4, TLR-2, and TLR-1) [24,25,26]. TLRs functioning endosomally are mainly activated with nucleic acids. On the other hand, a variety of molecules activate TLRs expressed on the cell surface. Most of them include lipoproteins. After ligand attachment, TLRs reorient domains and dimerize, activate intracellular cascade, and promote further cytoplasmatic signaling [27,28]. Immunotherapeutic agents aim at this activation chain, targeting immune-related disorders. Many autoregulating mechanisms control inflammation that is mediated through TLR signaling. Their activity concerns the nucleus level (countering the expression of cytokines and interleukins (TTP, ATF3, and REG-1) and the cytoplasmatic level (STAT1, AhR, Nurr1); the inhibition of adaptor complex suppressor of cytokine signaling, sterile alpha-and armadillo-motif-containing protein (SOCS1, SARM); and the cell-surface level interrupting dimerization processes: suppressor of tumorigenicity 2, single immunoglobulin IL-1R-related molecule, Rickettsia prowazekii 105 (ST2, SIGGR, and RP105) [29,30,31,32,33]. Moreover, some studies revealed microRNA molecules over activity destabilizing the mRNA of various cytokines. MiR-155-5p targets the MyD88 complex, similarly to MiR 203-5p and MiR 149-5p [30,34]. The described mechanisms of autocontrol ensure an adequate reaction to microbe-associated molecular patterns (MAMPs) and danger-associated molecular patterns (DAMPs), protecting autoimmunity and excessive response. TLR-4, after particular antigen attachment to a pathogen-associated molecular patterns (PAMP) or DAMP, is shifted from the cell surface to form the endosome during phagocytosis (Table 2). 

## 2. TLR-4 Overview

Toll receptors (TRs) were detected in drosophila embryos by Hashimoto et al. (1988) [35,36]. Hoffmann and their study team proved that TR mutation in drosophila raised the risk of fungal infection. The above experiments indicated that the innate immune response has the ability to precisely detect the invasion of bacteria and other micro-organisms [37]. Further studies identified mammalian TR homologs that researchers called toll-like receptors (TLRs). The first, discovered by Medzhitov R. and colleagues in 1997, was named TLR-4 [38,39]. The TLR-4 gene is localized on the SSC9:119.5, 9q32-33 chromosome, structured with three exons. The receptor is built of four domains and 838 amino acids. The first, the extracellular, consists of 624 amino acid molecules; the second, the transmembrane, consists of 33 amino acids; the third, the cytoplasmatic proximal, of 159 amino acids; and the cytoplasmatic distal of 19 amino acids. The ectodomain is built of 21 leucine-rich repeat regions (LRRs), part of the extracellular domain [40,41,42]. TLR-4 presents multiple polymorphism phenomena concerning single nucleotides (SNPs). Most of them, recognized in the ectodomain, promote detrimental phenotypic outcomes [43,44].

### 2.1. TLR-4 Signaling

To bind lipopolysaccharides, TLR-4 requires co-operating molecule myeloid differentiation 2 molecule (MD-2), extracellularly stabilizing the ligand during activation. After antigen ligation, the receptor dimerizes and activates TIR domains; to properly signal, TLRs require MyD88, an essential adaptor activated during the first stages after pattern ligation to promote an immune response. Activation of the transmembrane receptoral structure of TLR-4 transfers signaling in two major ways, the TIRAP–MyD88-dependent pathway controlling primary nuclear factor kappa-light-chain-enhancer of activated B cells (NF-κB) promotion, and the combined production of inflammatory cytokines. Activation of MyD88 results in IL-1R-associated kinase 1/TNFR-associated factor 6 (IRAK-1/TRAF-6) induction with collateral ubiquitination, and TRAF-6 promotes Transforming growth factor-beta-activated kinase 1 (TAK-1). Activation of TAK-1 results in the formation and promotion of the IKK/NF-κB complex. Mitogen-activated protein kinases-c-Jun. N-terminal kinase (MAPK-JNK) and extracellular signaling-regulated kinase (ERK1/2) kinases are also activated, which indicates activated protein-1 (Ap-1). Downsignaling promotes transcription factors, such as activated Ap1, stabilizing many genes with regulatory properties during inflammation [45,46,47].

**Figure 1:**
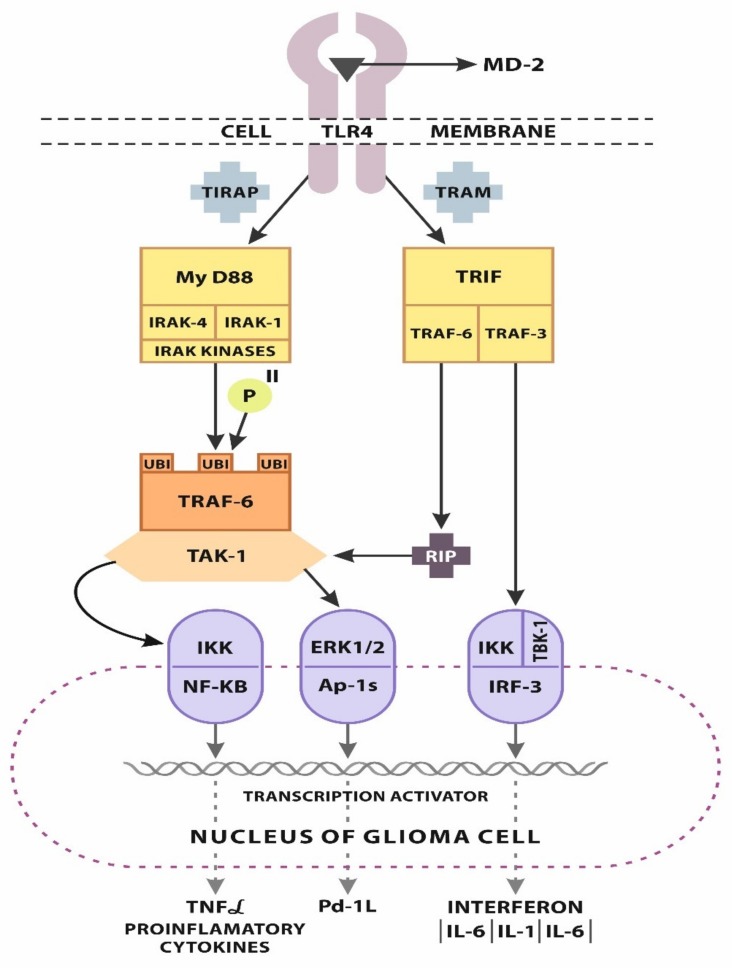
Toll-like-receptor (TLR)-4, after activation by pathogen-associated molecular patterns (PAMPs) and danger-associated molecular patterns (DAMPs) supported by myeloid differentiation factor-2 (MD-2), initiates downstream signaling in two major ways. The first is the MyD88-dependent pathway connected with toll-interleukin 1 receptor domain-containing adaptor protein (TIRAP) resulting in myddosome formation, a structure containing IRAK-4 and IRAK-1 kinases. Activation of IRAK-1 results in TRAF-6 induction with collateral ubiquitination and phosphorylation. TRAF-6 promotes TAK-1. Activation of TAK-1 results in IKK/NF-κB complex formation and promotion. ERK1/2 kinases are also activated, which indicates activated protein-1 (Ap-1), the factor activating the transcription process. The second pathway, MyD88 -independent, connected with translocating chain-associating membrane (TRAM), leads through toll-like receptor adaptor molecule-1 (TRIF) activation resulting in TRAF-3 and TRAF-6 promotion. RIP is recruited by TRAF-6, activating RIP signaling on the TAK-1/ERK 1/2/Ap-1 axis. TRAF-3 activates IKK and TBK-1 to activate interferon regulatory factor-3 (IRF-3). IRF-3 induces interferon expression and other proinflammatory cytokines.

The second toll-like receptor adaptor molecule-1-translocating chain-associating membrane (TRIF-TRAM) signaling pathway (MyD88-independent) activates interferon regulatory factor-3 (IRF-3). TRAM signaling, through TRIF activation, results in TRAF-3 and TRAF-6 promotion (Figure 1); RIP is recruited by TRAF-6, and RIP signaling is activated on TAK-1/ERK1/2/Ap-1 axis. TRAF-3 activates IKK and TBK-1 to activate IRF-3. IRF-3 as a transcription factor that upregulates genes, coding mainly the first types of IFNs and other proinflammatory cytokines. The MyD88-independent pathway stimulates TNFα secretion and production. The sequential ligation of TNFa to a proper receptor favors NF-κB promotion. Therefore, the MyD88-independent pathway induces the activation of NF-κB in a late phase via IRF-3 and the secretion of TNFα. Proinflammatory molecules, such as IFN, pro-IL-1, and pro-IL-6, are also promoted [48,49,50,51,52].

### 2.2. Progression of GBM

Numerous signaling pathways induce carcinogenesis, oncogenic transformation, and invasiveness of cancer cells. Interestingly, some of them promote NF-κB expression. NF-κB, as an important transcription factor, is involved in various cellular processes, including migration, proliferation, and survival. Inflammatory cytokines, PAMP and DAMP, could trigger further pro- NF-κB signaling throughout the activation of TNFRs and TLRs [53,54,55,56]. The deregulated activity of NF-κB is becoming an indicator of most neoplasm processes, including GBM. Evidence emphasizes TLRs induced carcinogenesis connected with NF-κB expression [57,58]. Kina et al. and Ferrandez et al., in studies concerning Glioma, confirmed the participation of the TLR-4/NF-κB pathway in the promotion of carcinogenesis [59,60]. Another interesting relation in glioma genesis is TLR-4/Programmed death receptor-1/Programmed death receptor-1 ligand (PD-1/PD-1L) axis interaction. Overexpression of TLR-4 results in an increase of PD-1L in GBM patients and is associated with unfavorable outcome [61]. Additionally, the increase inTLR-4 expression results in Wnt/claudine signaling forwarding to the progression of GBM and limits the effective apoptosis [62]. Progression of glioblastoma is also controlled and modulated by multiple miRNAs molecules. They control the cell circle of glioma cells on different stages of development. The invasiveness of GBM involves the change in epithelial cells polarity and loss of cell–cell adhesive properties. Down expression of E-cadherins results in modifications in the architecture of glioma cells, loss of integrity, and at last, tumor invasion. Increased expression of miR-10-b, miR-29, miR-146 accelerate invasion of GBM and predict an inauspicious outcome. Overexpression of miR-21 inhibits apoptosis and promotes GBM cell survival. miR 210-3p and miR- 93 promote angiogenesis. Moreover, miR 130b, miR 140, and miR 184 are intensively expressed in cases of histological progression of glioma [63,64,65,66,67,68,69,70]. TLR-4 signaling is modulated by multiple miRNAs molecules, and a great number of them inhibit this pathway. Xu et al. challenged the thesis that long noncoding RNA UBE2R2-AS1 targets TLR-4/miR 877-3p and promotes apoptosis and reduces invasiveness in glioma tissue. The study revealed that UBE2R2-AS1 targets miR 877-3p, inhibits its limiting effect on TLR-4, and as a result, promotes TLR-4 dependent apoptosis [71]. 

## 3. Potential Immunotherapeutic Targets

Developments made in neuroimmunology and tumor biology revealed interesting properties of TLR. Activation and TLR-4 downsignaling seem to play an interesting role in the promotion of glioma growth and invasion. On the other hand, some studies discovered that, in some special conditions, they could take part in the anticancer response. The release of HMGB1 from GBM dead cells during radio and chemotherapy stimulates the TLR4 pathway. It leads directly to Dendritic Cell maturation and efficient tumor antigen presentation leading to GBM regression in animal models [72,73,74,75,76,77]. There is adequate evidence clarifying that the modulant of TLR-4 proves efficiency and safety for GBM treatment based on previous trials concerning different neoplasms. The data imply that TLR-4 signaling has a dual disposition as a double-edged sword [61,78]. Taking control over this complex interplay could bring a satisfactory result in GBM matters. miRNAs, immune checkpoints, the Wnt axis, and glioma stem cells (SCS) could be used as a path to the complicated goal of effective glioblastoma therapy.

### 3.1. miRNA/TLR-4 Interplay

miRNAs represent a group of short noncoding strands of RNA with length oscillating 20–22 nucleotides each. Around 30% of all genes are controlled and regulated by miRNAs. Molecules bind to the targeted mRNA with the 3′ region called the untranslated (UTR) monitoring the expression of particular genes through degradation or translation breakdown. As a result, miRNAs are engaged in various biological processes, such as homeostasis, growth, differentiation, immune activation, apoptosis, and stress response. Otherwise, they modulate the immune response to pathogen invasion throughout the TLR signaling pathway (modulation of cytokines, transcription factors, and signaling proteins and receptors). Additionally, miRNA activity is similarly detected in normal healthy tissue in chronic diseases and cancers [79,80,81,82,83,84,85,86,87].

Levels of miRNAs are significantly higher in GBM tissue and GBM cell lines [87,88,89,90,91,92]. The impact of those molecules in carcinogenesis is still unclear, requiring further investigation [93]. There is evidence pointing at a strong relation between TLR-4 signaling and miRNA expression. TLR-4/miRNA interplay changes the typical balance between immune response and inhibition. The indirect influence of mRNAs concerning TLR downsignaling was represented by the study by Taganov et al. The study team documented lipopolysaccharide-induced expression of miR-132, miR-155, and miR 146a, regulating TLR downsignaling [94,95,96], Another study connected miR-34a expression with significant antitumor properties in glioma p-53 mutant cell line U251. Overexpression of miR-34a induces apoptosis and inhibits the growth of glioma through the activation of numerous particles. A similar effect of miR-34a was revealed by Xu et al. while investigating breast cancer. In their study, the miR-34a molecule was found to inhibit C-X-C motif ligand 10 (CXCL10). The ligand is characterized by a strong affinity to TLR, promoting cancerogenic downsignaling [97,98]. Indirect inhibition of TLR signaling results in the regression of tumor growth and expansion. Direct TLR-4 inhibition is induced by different miRNAs [99,100,101,102]. miRNA molecules directly develop inhibitory features, in particular during stages of TLR-4 activation. The MyD-88-dependent pathway IRAK1–TRAF6 complex is inhibited by miR 93-5p, miR 302b-5p, miR 124-5p, and miR 146a/b-5p. The TAK-1 complex is inhibited by miR 23b-5p, miR 142-3p, miR 155-5p, and miR 23-5p. Signaling through the Myd88-independent pathway is regulated by miR3178-5p and miR 3473-5p as inhibitors of TRAF-3, and miR 21b-3p, miR 146a-5p, and miR 302c-5p as direct inhibitors of IRF-3 [94,103,104,105,106,107,108]. TNF-alfa is inhibited by miR 19a-5p, miR125b-5p, and miR 203-5p. IL-6 is blocked by miR 9-5p, miR26a-5p, miR100-5p, and miR 365, and IL-10 is inhibited by miR-98-5p and miR-106a-5p [109,110].

### 3.2. Immune Checkpoints vs. TLR-4

Immune checkpoints (ICs) are molecules with the ability to modulate T cells. Coinhibitory and costimulatory properties ensure a balanced immune response. ICs protect autoimmunity and optimize the adequate response of the immune system. When carcinogenesis occurs, IC signaling becomes a route of immune escape, leading to aggressive tumor invasion. Cytotoxic T-lymphocyte antigen 4 (CTLA-4) was the first well-described IC molecule expressed on CD-8+ and CD-4+ T cells. CTLA-4 binds to the corresponding B7-1 (CD80) ligand on antigen-presenting cells (APCs). Interaction between both results in the inhibition of T cells. In GBM, CTLA-4 expression on CD8+ and CD4+ has a significant correlation with poor outcome and invasion of brain tissue. TLR signaling promotes the overexpression of CD80, enhancing the presentation of antigens. It strengthens CTLR-4-induced T-cell anergy and GBM expansion [111,112,113,114].

The PD-1/PD-1L axis is another IC pathway. PD-1 and PD-1L are the most potent immunomodulatory proteins. PD-1L was described in 1999 by the Dong study team as a B7-H1 molecule [115], which represents the B7 protein family with a homology level of around 20%. The expression of PD-1L could be divided into inducible and constitutive. The constitutive can be met on antigen-presenting cells and resting lymphocytes. Inducible expression appears during inflammation and immune response; PD-1L represents suppressive properties. Glioblastoma cells represent both constitutive and inducible expression of PD-1L [116,117]. The phenomenon of autoinduction through TLR-4 also occurs. TLR-4 activation on GBM results in signaling through the MyD-88-independent pathway, TRAF6/ERK-1/2 AP-1s, leading to the activation of the PD-1L promoter. PD-1L mRNA is translated into ribosomes, modified in the Golgi apparatus, and presented as a mature PD-1L forming on the surface of GBM cells. T cells recognize PD-1L and bind it with an adequate PD-1 receptor. The intracellular domain of the receptor consists of an (ITSM) tyrosine-based switch motif that activates an inhibitory cascade [118,119,120,121,122,123,124]. Downsignaling promotes tyrosine phosphatase 2/Zeta-chain-associated protein kinase 7 (SHP-2/Zap 70) interplay, resulting in dephosphorylation and a significant decrease in lymphocytic cytotoxicity and proliferation. Activation of the PD-1/PD-1L axis leads to T-cell anergy and increases the lymphocyte apoptotic ratio. Lipopolysaccharides (LPsS), high-mobility group box-1 proteins (HMGB1), and the heat-shock-protein (HSP) family act as TLR-4 agonists that initiate signaling promoting PD-1L expression. Most of those molecules are intensively expressed in the GBM microenvironment, resulting in the autocrine induction of regional immunosuppression controlled by the PD-1L/PD-1 axis [125,126,127].

A study performed by Beswick et al. [128] discussed the matter of TLR-4/PD1L-induced immunotolerance in colon mucosa. Additionally, Wolfle et al. described a similar inductive effect of TLR-4 activation on PD-1L expression [129]. Poor prognosis for patients with peripheral lymphomas, correlated with TLR-4 and PD-1L overexpression, presented by Zhao et al., confirmed the pro-oncogenic effect of the presented axis [130].

### 3.3. TLR-4/Wnt Axis and Apoptosis

Glioblastoma, as a highly aggressive neoplasm, activates or modulates many pathways promoting gliomagenesis. One of those downsignaling instances concerns wingless signaling (Wnt\claudines axis) occurring as a crucial controller of cell–cell interplay, and as a regulator of migration. Any abnormalities in Wnt lead to evolutive effects. Aberrations in Wnt balance between stimulation and inhibition usually promote carcinogenesis. The Wnt complex represents an extended network consisting of various components, crosstalk interactions, and multiple regulatory stages. Wnt usually starts with receptor and coreceptor stimulation. Receptors, such as low-density lipoprotein receptor-related protein 6 (LRP6), protein Tyr kinase 7 receptor (PTK7), Tyr kinase-like orphan receptor (ROR), skeletal muscle Tyr kinase receptor (MUSK), and Tyr kinase receptor (RYK), are treated as Wnt-related. The main regulation of receptors is intracellularly processed by phosphorylating kinases. Extracellular regulation takes place through active agonists, such as norrins, the R-spondin family, and antagonists WNT inhibitory factor (WIF), secreted frizzled-related protein (SFRP), sclerostin, and Dickkopf-related protein 1 (DKK1) [131,132,133,134,135,136,137].

Wnt pathway activation is connected with other molecules called claudins. Claudins are a family of proteins that determine tight junctions (TJs) (claudin-5, claudin-3, and claudin-1). Claudin-5 is mainly expressed on the lung epithelium and brain tissue. Claudin-5, built-in TJs, bands in CNS endothelial cells. TJs create direct intercellular adhesion reducing cell-to-cell distance. In effect, they form the real connection between particular endothelial cells, strengthen the integrity of vessels, and control the diffusion of different ions and solutions. CNS embryonal development changes the permeability ratio. An immature form of the CNS vasculature, leaky blood vessels with characteristic fenestrations, are replaced by more integrating TJs. Claudin-5 contributes to the function of the blood barrier [138,139,140,141,142]. Overexpression of claudin-5 reduced inulin diffusion through the brain-vessel endothelium in rat models [143]. On the other hand, the downexpression of claudin-5 promotes epithelial permeability and the loss of blood–brain-barrier (BBB) properties [144]. Lower levels of claudin are correlated with higher levels of TLR-4 expression [145,146,147,148].

Dickkopf-related protein-3 (DKK-3), as was mentioned above, represents a group of proteins with strong suppressive features able to halt Wnt signaling. Moreover, DKK-3 seems to be an effective inhibitor of tumor-cell growth molecules. Casili et al. performed a study revealing DKK-3 to have a regulating influence on the Wnt pathway, resulting in increased caspase-3-dependent apoptosis [62].

Caspase-3, a representative of the caspase family, is described as an effector caspase. Its properties, such as activation by many apoptotic inducers, result in rife activity leading to complete apoptosis in many cell lines. Overexpression of caspase-3 results in an apoptotic GBM phenotype, confirmed in patient samples. Studies suggested that a higher apoptotic ratio is associated with longer PFS in treating for GBM. Sensitivity to temozolomide (TMZ) treatment is also correlated with caspase-3 expression, and higher levels of caspase-3 promote TMZ efficiency. Interestingly, caspase-9, a less apoptotic molecule, is downexpressed after Dickkopf WNT Signaling Pathway Inhibitor 3 (DKK-3) upregulation, usually increasing after TLR-4 stimulation. Highly invasive cancer-cell lines express caspase-9 more intensively [62,149,150,151].

Apoptosis, as a canonical biological interaction, regulates the subsistence of organisms and limits the expansion of GBM. A study connected the expression of DKK-3 and claudine-5 in healthy brain tissue, compared with the downexpression of both in GBM patients. Significantly lower levels of DKK-3 were observed in many types of neoplasms concerning prostate cancer. The absence of TLR-4 inhibits the growth of glioblastoma lines. The expression of TLR-4 interferes with Wnt downsignaling, promoting carcinogenesis. Additionally, the lack of TLR-4 maintains claudin-5 and DKK-3 proapoptotic properties, resulting in the limitation of tumor expansion. To sum up, the downexpression of TLR-4 promotes Wnt/DKK-3/claudin-5 signaling, which limits GBM invasiveness [62,152,153].

### 3.4. TLR-4 Influence on Non-CSC Glioma Stem Cells

A study performed by Alvarado et al. revealed a lack of TLR-4 occurrence on cancer stem cells (CSCs), the most invasive and most aggressive subpopulations of GBM. In opposition to CSC mature GBM cells and non-CSCs, it demonstrated TLR-4 expression and a response to agonist ligation. CSCs are responsible for therapeutic ineffectiveness and tumor-mass progression. External stimulation, such as necrosis, hypoxia, uncontrolled proliferation, and acidic stress, characteristics of a tumor microenvironment, generate unfavorable conditions. Thanks to developed adaptive mechanisms, CSCs maintain the balance between self-restoration and differentiation in hostile habitats. Owing to limited TLR-4 expression, CSCs can survive in a malevolent environment. Experiments conducted by a study team explained the processes of suppressing the CSC subpopulation, in which TLR-4 signaling played a key role. The main discovery in those papers indicated intensive TLR-4 downsignaling, halting CSC expansion by reducing the activity of retinoblastoma binding protein 5 (RBBP5), which is naturally increased in this subpopulation. Another important thing to understand in the underlying processes in CSCs is that RBBP-5 promotes cardinal stem-cell transcription initiators, essential to achieve invasiveness and self-restoration. TLR-4 expression and downsignaling inhibit RBBP-5 by the phosphorylation of TBK-1. TLR signaling borders proliferative trends in CSC and GBM [154,155,156,157,158,159,160].

**Figure 2:**
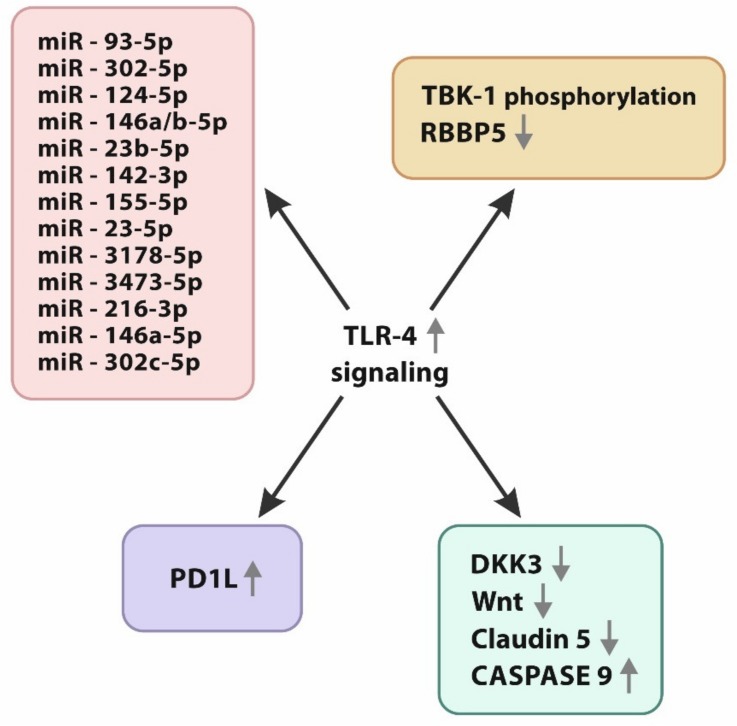
Potential targets for future immunotherapy concerning TLR-4 signaling in glioblastoma multiforme: Complex of miRNA molecules inhibiting TLR-4 signaling on different stages, programmed death ligand-1 (PD-1L) overexpression stimulated by TLR downsignaling, indirect retinoblastoma binding protein 5 (RBBP5) inhibition by TBK-1 phosphorylation resulting in antineoplastic activity, interplay between TLR-4 and Dickkopf-related protein 3 (DKK-3) inhibition, Wnt pathway signaling restriction, claudin-5 level reduction, and caspase-9 elevation and caspase-3 decrease as a pro-oncogenic cascade after TLR-4 activation.

Recent studies concerning bladder tumors, prostate cancer, and colorectal cancer pointed at TLR signaling as a tumorigenic and tumor-progression factor [161,162,163,164]. Dapito et al. linked TLR-4 activation with the significant progression of hepatocellular carcinoma. Alvarado et al. presented the opposite effects, where the receptor limited tumor growth by the direct blockade of the self-restoration cycle. The described divergence of TLR-4 signaling could be linked with ligand properties and downsignaling; TBK-1 seems to be an explanation, which is an element of the TLR-4–MyD88-independent pathway, and activation may also be signaled through the MyD88-dependent pathway, as it occurs in hepatocellular cancer [160,165]. Further observation and experiments explaining these differences are required, which are necessary to understand the anti- and procarcinogenic features of TLR-4. Signaling through the MyD88-independent and MyD88-dependent pathway may bring totally different results. Elucidation of these opposite results is crucial for future perspectives of immunotherapy for GBM (Figure 2).

## 4. Trials Concerning TLR-4

### 4.1. In Vitro and In Vivo Agonist

Exposition of TLR-4 on LPS in in vitro studies resulted in tumor-cell-line progression. The promotion and proliferation of the U87 and U118 lines were detected, and an increase in invasion was realized in U87 [28]. Stimulation of LPS increased metalloproteinase-9 (MMP-9), a factor of invasion detected in U87 glioma cell lines. Moreover, parallel TLR-4 signaling modulated the evasion of the immune system, tumor-necrosis-factor resistance, and cell survival [166]. The spirulina complex (polysaccharide) and LPSs as TLR-4 agonists demonstrated antitumor activity in mouse models. Differences in the action of both were underlain in IL-17 interaction. IL-17, with its pleiotropic characteristic, acts as an anti- or protumor cytokine, depending on the investigated neoplasm model. An increase in the spirulina polysaccharide, in contrast to LPS, increased the levels of IL17 in mouse-model serum. The murine glioma-cell complex becomes more invasive and expands after IL-17 activation [167,168]. Moreover, Fas and TLR-4 contemporary activation results in the disappearance of TLR-4 tumor-promoting properties [169].

Prosaposin (PSAP) conserved glycoprotein acts as a neurotropic factor in the glioblastoma environment. High levels of PSAP were detected in glioblastoma patients and were associated with unfavorable outcome [27,170,171]. Jiang et al. investigated PSAP as a promotor of proliferation and tumorigenesis through TLR-4 signaling. Mice models confirmed poor prognosis related to PSAP overexpression. The study hypothesizes PSAP affinity to TLR-4. Co-localization of TLR-4 and PSAP was confirmed with immunofluorescence staining. To sum up, PSAP activates TLR-4/NF-κB signaling, induces secretion of factors responsible for inflammation and tumor growth [172]. The blockade of PSAP becomes an interesting target for improving GBM outcome.

LPS was examined as affecting GSCs, and mature glioma cells in the study performed by Han et al. rat model has proven that LPS stimulation prolonged survival time significantly in the observed glioma-cohort [173]. Interestingly some studies report an antitumoral effect induced by a bacterial infection in animal models and report some cases of GBM patients [174,175,176,177]. LPS via TLR-4 stimulation changes immuno-phenotype in GSCs and the mature form of glioma cells and induces antitumoral response [173]. 

### 4.2. Agonist and Inhibitors of TLR-4 in Oncology

TLR-4 is expressed on the surface of the cell membrane and occurs on endosomes. Moreover, the receptor has the ability to dependently signal to MyD88 and TRIF complexes. Those characteristic features are the example of evolutionary controlled carefulness in precise antigen-detection and downsignaling-promotion mechanisms. TLR-4 coreceptor myeloid differentiation factor 2 (MD-2) has an appropriate pocket that binds the corresponding ligands. Immunotherapy disrupting TLR-4 and MD-2 binding or blocking their interplay was explored [178,179,180]. Another interesting ligand inhibiting or activating TLR-4, containing a glucopyranosyl lipid adjuvant (GLA) agonist [181,182], monophosphoryl lipid A (MPLA) agonist [183,184], and lipid 4A antagonist, was discovered and explored [185]. Interestingly, TLR-4 is the most popular among the TLRs evaluated as receptors in numerous clinical trials as a potential target for immunotherapy against various pathologies concerning inflammation, viral infection, immune diseases, and cancers (Table 3) [186,187,188].

### 4.3. TLR-4 in Glioblastoma Multiforme—Clinical Application

The macrophage migration inhibitory factor (MIF) cytokine is treated as a potent cytokine initiating immunity and inflammation processes [189,190,191]. MIF is excreted by macrophages immediately after the ligation of bacterial products and inflammatory molecules. Studies showed a strict interplay between MIF and TLR-4 signaling. MIF knockout mice presented the downregulation of TLR-4 signaling. The response of macrophages to LPS stimulation was significantly limited [192,193,194,195]. The presented model made the basis for immunotherapy targeting MIF and TLR-4. Trial NCT03782415 contested ibudilast MN-166 in Phase 1 and compared it with TMZ combo treatment in recurrent GBM in Phase 2. A multicenter open-label study evaluated its efficacy, tolerability, and safety with a TMZ combination in 50 recurrent GBM patients. To be eligible, patients required a Karnofsky Performance Scale (KFS) > 70 (https://clinicaltrials.gov/ct2/show/NCT03782415).

Ibudilast is an agent that is able to diffuse the blood–brain barrier (BBB), developing antitumor activity by inhibiting MIF and phosphodiesterase-4 (PDE-4), also reducing TLR-4 downsignaling with pro-oncogenic properties. Action leads to excessive apoptosis and the limitation of cell proliferation in GBM [196,197,198]. Ibudilast was also observed in trials, including neuroinflammation, amyotrophic lateral sclerosis (ALS), dysphoria, treatment of alcohol-use disorders, and methamphetamine dependence (https://clinicaltrials.gov/ct2/results?cond=ibudilast&term=&cntry=&state=&city=&dist=).

Another clinical application of TLR-4 signaling concerns the antiglioma HSP vaccination. HSP, as a part of the heat shock protein peptide complex (HSSPC) (Table 4), is characterized by a strong affinity to numerous receptors on the surface of the cell. T-cell supported HSP stimulation of APCs results in downstream signaling and NF-κB activation. CD91/CD40/CD36/CD14/TLR-2 and TLR-4 seem to play crucial roles in this process. Promotion of the TLR-4/NF-κB axis in APC causes intensive secretion of chemokines and proinflammatory molecules to the tumor microenvironment. Ipso facto, HSP stimulates, indirectly, the release of inflammatory factors, such as IL-12, IL-1beta, TNF alfa, granulocyte macrophage colony-stimulating factor (GM-CSF) and inhibits GBM growth [199,200,201,202,203,204,205,206].

The study performed by Crane et al. [207] reported significant immunization after HSP administration in 11 out of 12 patients with recurrent GBM (rGBM). Median Survival time in the group of immune responders oscillated about 47 weeks after surgical resection and vaccination. Non-responding patients survived 16 weeks only. Promising results encouraged for further investigation. Bloch et al. revealed a median overall survival oscillating 42 weeks (42.6 weeks) after HSPPC-96 administration in rGBM patients. Further efforts concerning the design of Phase2 studies have been performed [208]. Clinical trials challenging the effectiveness and safety of HSPPC-96 vaccination evaluate overall survival and progression-free time. Trials also compare results with standard treatment, other immunological agents (Bevacizumab, Pembrolizumab), and the combination of drugs (https://www.clinicaltrials.gov) (Table 4).

## 5. Conclusions

TLR-4 signaling pathways and their wide spectrum of interactions seem to be promising targets for immunotherapy. Multimodal interplay and a variety of downsignaling encourage TLR-4 to be further considered as a potential aim for immune agents. Clinical trials contesting TLR-4 agonists and inhibitors are recruiting patients with various cancers. TLR-4-related treatment is being gradually introduced for GBM patients. There is still much to do in the field of effective immunotherapy.

## Figures and Tables

**Table 1:** Toll-like-receptor (TLR)-4 expression in brain tissue, cancer lines, and tumors.

TLR-4 Expression	Localization	Type of Cells
mRNA/protein	Tissue	Neurons, microglia [13,14,15]
mRNA/protein	Tissue	Astrocytes [16,17]
mRNA	Cell Lines	Glioma (U87, SF126, U251, GI 261) [18]
mRNA/protein	Tumor/Cell Lines	Astrocytoma/GBM (U87MG, A172, LN229, U118) [19]

**Table 2:** Characteristics of TLR-4 receptor: types of cells expressing TLR-4, pathogen-associated molecular patterns (PAMPs), danger-associated molecular patterns (DAMPs), and clinical-trial agents.

TLR-4			
Types of Cells	PAMPs	DAMPs	Clinical-Trial Agent Characteristics
Monocytes Macrophages Neutrophil Myeloid dendritic cells Mast cells B cells Intestinal epithelium Platelets	Lipopolysaccharide	HSPs, heparin, fibrinogen, fibronectin, sulfate, HMGB1, ANG II	Anti-TLR-4 antibody, lipid A derivates, polysaccharides.

**Table 3:** First- and second-phase clinical trials concerning TLR-4 agonists and antagonists in the treatment of various neoplasms.

Clinical Trial Number	Phase	Indication	Agonist/Antagonist of TLR-4	Ligand Characteristic
NCT 02320305	1	Melanoma	Agonist	MART-1
NCT 02180698	1	Soft tissue sarcoma	Agonist	GLA-SE
NCT02501473	1,2	Follicular lymphoma	Agonist	G100
NCT02995655	1	Acute myeloid leukemia	Antagonist	CX100
NCT02035657	1	Merkel cell carcinoma	Agonist	GLA-SE
NCT02270372	1	Breast and ovarian cancer	Agonist	ONT-10
NCT01556789	1	Solid tumors	Agonist	ONT-10
NCT02609984	2	Sarcoma	Agonist	CMB-305
NCT02387125	2	Non-small lung cancer	Agonist	CMB-305

**Table 4:** Clinical trials concerning heat shock protein peptide complex (HSSPC) vaccination in glioblastoma-multiforme (GBM) patients. (https://www.clinicaltrials.gov).

Clinical Trial Number	Phase	Immunological Agent	Number of Participants	Indication	Age	Country
**NCT03650257**	2	Autologous Heat Shock Protein (gp96) Vaccine	150	GBM	Adult	China
**NCT02722512**	2	HSPPC-96 Vaccine	20	hGG, rGBM, GBM, Ependymoma	Child/Adult	USA
**NCT03018288**	22	Pembrolizumab (anti PD-1), HSPPC-96 Vaccine	108	GBM	Adult	USA
**NCT00905060**	2	HSPPC-96 Vaccine	46	GBM	Adult	USA
**NCT00293423**	1/2	HSPPC-96 Vaccine	41	rGBM	Adult	USA
**NCT01814813**	2	HSPPC-96 Vaccine with Bevacizumab	90	rGBM	Adult	USA

## References

[1] Lee H., Lee S., Cho I.H., Lee S.J. (2013). Toll-like receptors: Sensor molecules for detecting damage to the nervous system. Curr. Protein Pept. Sci..

[2] Tewari R., Choudhury S.R., Ghosh S., Mehta V.S., Sen E. (2012). Involvement of TNFα-induced TLR4-NF-κB and TLR4-HIF-1α feed-forward loops in the regulation of inflammatory responses in glioma. J. Mol. Med. (Berl.).

[3] Zeuner M., Bieback K., Widera D. (2015). Controversial role of tolllike receptor 4 in adult stem cells. Stem Cell Rev..

[4] Ulrich H., do Nascimento I.C., Bocsi J., Tárnok A. (2014). Immunomodulation in stem cell differentiation into neurons and brain repair. Stem Cell Rev..

[5] Mazurek M., Litak J., Kamieniak P., Kulesza B., Jonak K., Baj J., Grochowski C. (2020). Metformin as Potential Therapy for High-Grade Glioma. Cancers.

[6] Huang B., Zhao J., Unkeless J.C., Feng Z.H., Xiong H. (2008). TLR signaling by tumor and immune cells: A double-edged sword. Oncogene.

[7] Su X., Ye J., Hsueh E.C., Zhang Y., Hoft D.F., Peng G. (2010). Tumor microenvironments direct the recruitment and expansion of human Th17 cells. J. Immunol..

[8] Ye J., Ma C., Hsueh E.C., Dou J., Mo W., Liu S., Han B., Huang Y., Zhang Y., Varvares M.A. (2014). TLR8 signaling enhances tumor immunity by preventing tumor-induced T-cell senescence. EMBO Mol. Med..

[9] Krawczyk C.M., Holowka T., Sun J., Blagih J., Amiel E., DeBerardinis R.J., Cross J.R., Jung E., Thompson C.B., Jones R.G. (2010). Toll-like receptor-induced changes in glycolytic metabolism regulate dendritic cell activation. Blood.

[10] Gil-Benso R., Monteagudo C., Cerdá-Nicolás M., Callaghan R.C., Pinto S., Martínez-Romero A., Pellín-Carcelén A., San-Miguel T., Cigudosa J.C., López-Ginés C. (2012). Characterization of a new human melanoma cell line with CD133 expression. Hum. Cell.

[11] Jackson M., Hassiotou F., Nowak A. (2015). Glioblastoma stem-like cells: At the root of tumor recurrence and a therapeutic target. Carcinogenesis.

[12] Nagyoszi P., Wilhelm I., Farkas A.E., Fazakas C., Dung N.T., Hasko J., Krizbai I.A. (2010). Expression and regulation of toll-likereceptors in cerebral endothelial cells. Neurochem. Int..

[13] Hanke M.L., Kielian T. (2011). Toll-like receptors in health and disease in the brain: Mechanisms and therapeutic potential. Clin. Sci..

[14] Bsibsi M., Ravid R., Gveric D., van Noort J.M. (2002). Broad expression of Toll-like receptors in the human central nervous system. J. Neuropathol. Exp. Neurol..

[15] Hussain S.F., Yang D., Suki D., Aldape K., Grimm E., Heimberger A.B. (2006). The role of human glioma-infiltrating microglia/macrophages in mediating antitumor immune responses. Neuro-Oncol..

[16] Bowman C.C., Rasley A., Tranguch S.L., Marriott I. (2003). Cultured astrocytes express toll-like receptors for bacterial products. Glia.

[17] Crack P.J., Bray P.J. (2007). Toll-like receptors in the brain and their potential roles in neuropathology. Immunol. Cell Biol..

[18] Hu J., Shi B., Liu X., Jiang M., Yuan C., Jiang B., Song Y., Zeng Y., Wang G. (2018). The activation of Toll-like receptor 4 reverses tumor differentiation in human glioma U251 cells via Notch pathway. Int. Immunopharmacol..

[19] Deng S., Zhu S., Qiao Y., Liu Y.J., Chen W., Zhao G., Chen J. (2014). Recent advances in the role of toll-like receptors and TLR agonists in immunotherapy for human glioma. Protein Cell.

[20] Kawai T., Akira S. (2009). The roles of TLRs, RLRs and NLRs in pathogen recognition. Int. Immunol..

[21] Akira S., Uematsu S., Takeuchi O. (2006). Pathogen recognition and innate immunity. Cell.

[22] Bianchi M.E. (2007). DAMPs, PAMPs and alarmins: All we need to know about danger. J. Leukoc. Biol..

[23] Krysko D.V., Garg A.D., Kaczmarek A., Krysko O., Agostinis P., Vandenabeele P. (2012). Immunogenic cell death and DAMPs in cancer therapy. Nat. Rev. Cancer.

[24] Meng Y., Kujas M., Marie Y., Paris S., Thillet J., Delattre J.-Y., Carpentier A.F. (2008). Expression of TLR9 withinhuman glioblastoma. J. Neuro-Oncol..

[25] Medvedev A.E., Sabroe I., Hasday J.D., Vogel S.N. (2006). Invited review: Tolerance to microbial TLR ligands:Molecular mechanisms and relevance to disease. J. Endotoxin Res..

[26] Aalaei-Andabili S.H., Rezaei N. (2013). Toll like receptor (TLR)-induced differential expression of microRNAs(MiRs) and immune response against infection: A systematic review. J. Infect..

[27] Han S., Wang C., Qin X., Xia J., Wu A. (2017). LPS alters the immuno-phenotype of glioma and glioma stem-like cells and induces in vivo antitumor immunity via TLR4. J. Exp. Clin. Cancer Res..

[28] Sarrazy V., Vedrenne N., Billet F., Bordeau N., Lepreux S., Vital A., Jauberteau M.O., Desmouliere A. (2011). TLR4 signal transduction pathways neutralize the effect of Fas signals on glioblastoma cell proliferation and migration. Cancer Lett..

[29] Han C., Jin J., Xu S., Liu H., Li N., Cao X. (2010). Integrin CD11b negatively regulates TLR-triggered inflammatory responses by activating Syk and promoting degradation of MyD88 and TRIF via Cbl-b. Nat. Immunol..

[30] Anwar M.A., Basith S., Choi S. (2013). Negative regulatory approaches to the attenuation of Toll-like receptor signaling. Exp. Mol. Med..

[31] Skaug B., Chen J., Du F., He J., Ma A., Chen Z.J. (2011). Direct, noncatalytic mechanism of IKK inhibition by A20. Mol. Cell.

[32] Hayden M.S., Ghosh S. (2012). NF-kappaB, the first quarter-century: Remarkable progress and outstanding questions. Genes Dev..

[33] Palsson Mc Dermott E.M., Doyle S.L., McGettrick A.F., Hardy M., Husebye H., Banahan K., Gong M., Golenbock D., Espevik T., O’Neill L.A.J. (2009). TAG, a splice variant of the adaptor TRAM, negatively regulates the adaptor MyD88independent TLR4 pathway. Nat. Immunol..

[34] Spachidou M.P., Bourazopoulou E., Maratheftis C.I., Kapsogeorgou E.K., Moutsopoulos H.M., Tzioufas A.G., Manoussakis M.N. (2007). Expression of functional Toll-like receptors by salivary gland epithelial cells: Increased mRNA expression in cells derived from patients with primary Sjogren’s syndrome. Clin. Exp. Immunol..

[35] Hashimoto C., Hudson K.L., Anderson K.V. (1988). The Toll gene of Drosophila, required for dorsal-ventral embryonic polarity, appears to encode a transmembrane protein. Cell.

[36] Winans K.A., Hashimoto C. (1995). Ventralization of the Drosophila embryo by deletion of extracellular leucine-rich repeats in the Toll protein. Mol. Biol. Cell.

[37] Hoffmann J.A. (1995). Innate immunity of insects. Curr. Opin. Immunol..

[38] Medzhitov R., Preston-Hurlburt P., Janeway C.A. (1997). A human homologue of the Drosophila toll protein signals activation of adaptive immunity. Nature.

[39] Medzhitov R., Janeway C.A.J. (1997). Innate immunity: The virtues of a nonclonal system of recognition. Cell.

[40] Randow F., Seed B. (2001). Endoplasmic reticulum chaperone gp96 is required for innate immunity but not cell viability. Nat. Cell Biol..

[41] De Nardo D. (2015). Toll-like receptors: Activation, signalling and transcriptionalmodulation. Cytokine.

[42] Kawai T., Akira S. (2010). The role of pattern-recognition receptors in innate immunity: Update on toll-like receptors. Nat. Immunol..

[43] Gao D., Li W. (2017). Structures and recognition modes of toll-like receptors. Proteins Struct. Funct. Bioinform..

[44] Uenishi H., Shinkai H. (2009). Porcine toll-like receptors:the front line of pathogen monitoring and possible implications for disease resistance. Dev. Comp. Immunol..

[45] Viriyakosol S., Tobias P.S., Kitchens R.L., Kirkland T.N. (2001). MD-2 binds to bacterial lipopolysaccharide. J. Biol. Chem..

[46] Ohto U., Fukase K., Miyake K., Shimizu T. (2012). Structural basis of species-specific endotoxin sensing by innate immune receptor TLR4/MD-2. Proc. Natl. Acad. Sci. USA.

[47] Shimazu R., Akashi S., Ogata H., Nagai Y., Fukudome K., Miyake K., Kimoto M. (1999). MD-2, a molecule that confers lipopolysaccharide responsiveness on toll-like receptor 4. J. Exp. Med..

[48] Kim H.M., Park B.S., Kim J.-I., Kim S.E., Lee J., Oh S.C., Enkhbayar P., Matsushima M., Lee H., Yoo O.J. (2007). Crystal structure of the TLR4-MD-2 complex with bound endotoxin antagonist eritoran. Cell.

[49] O’Neill L.A., Bowie A.G. (2007). The family of five: TIR-domaincontaining adaptors in Toll-like receptor signalling. Nat. Rev. Immunol..

[50] Park B.S., Song D.H., Kim H.M., Choi B.-S., Lee H., Lee J.-O. (2009). The structural basis of lipopolysaccharide recognition by the TLR4-MD-2 complex. Nature.

[51] Zeuner M.T., Kruger C.L., Volk K., Bieback K., Graeme C.S., Heilemann M., Widera D. (2016). Biased signalling is an essential feature of TLR4 in glioma cells. Biochim. Biophys. Acta.

[52] Finocchiaro G. (2017). TLRgeting evasion of immune pathways in glioblastoma. Cell Stem Cell.

[53] Park M.H., Hong J.T. (2016). Roles of NF-jB in cancer and inflammatory diseases and their therapeutic approaches. Cells.

[54] Soubannier V., Stifani S. (2017). NF-κB Signalling in Glioblastoma. Biomedicines.

[55] Pikarsky E., Porat R.M., Stein I., Abramovitch R., Amit S., Kasem S., Gutkovich-Pyest E., Urieli-Shoval S., Galun E., Ben-Neriah Y. (2004). NF-kappaB functions as a tumour promoter in inflammation-associated cancer. Nature.

[56] Ditsworth D., Zong W.X. (2004). NF-kappaB: Key mediator of inflammationassociated cancer. Cancer Biol. Ther..

[57] Kına I., Sultuybek G.K., Soydas T., Yenmis G., Biceroglu H., Dirican A., Uzan M., Ulutin T. (2018). Variations in Toll-like receptor and nuclear factor-kappa B genes and the risk of glioma. Br. J. Neurosurg..

[58] Hanahan D., Weinberg R. (2011). Hallmarks of cancer: The next generation. Cell.

[59] Harazono Y.L., Muramatsu T., Endo H., Uzawa N., Kawano T., Harada K., Inazawa J., Kozaki K. (2013). miR- 655 Is an EMT-Suppressive MicroRNA Targeting ZEB1 and TGFBR2. PLoS ONE.

[60] Gabriely G., Wurdinger T., Kesari S., Esau C.C., Burchard J., Linsley P.S., Krichevsky A.M. (2008). MicroRNA 21 promotes glioma invasion by targeting matrix metalloproteinase regulators. Mol. Cell Biol..

[61] Chaudhary D., Robinson S., Romero D.L. (2015). Recent advances in the discovery of small molecule inhibitors of interleukin-1 receptor-associated kinase 4 (IRAK4) as a therapeutic target for inflammation and oncology disorders. J. Med. Chem..

[62] Casili G., Caffo M., Campolo M., Barresi V., Caruso G., Cardali S.M., Lanza M., Mallamace R., Filippone A., Conti A. (2018). TLR-4/Wnt modulation as new therapeutic strategy in the treatment of glioblastomas. Oncotarget.

[63] Li Y., Wang Y., Yu L., Sun C., Cheng D., Yu S., Wang Q., Yan Y., Kang C., Jin S. (2013). miR-146b-5p inhibits glioma migration and invasion by targeting MMP16. Cancer Lett..

[64] Cui B., Li B., Liu Q., Cui Y. (2017). lncRNA CCAT1 Promotes Glioma Tumorigenesis by Sponging miR-181b. J. Cell Biochem..

[65] Sun L., Yan W., Wang Y., Sun G., Luo H., Zhang J., Wang X., You Y., Yang Z., Liu N. (2011). MicroRNA-10b induces glioma cell invasion by modulating MMP-14 and uPAR expression via HOXD10. Brain Res..

[66] Wu D.G., Wang Y.Y., Fan L.G., Luo H., Han B., Sun L.H., Wang X.F., Zhang J.X., Cao L., Wang X.R. (2011). MicroRNA-7 regulates glioblastoma cell invasion via targeting focal adhesion kinase expression. Chin. Med. J. (Engl.).

[67] Lin J., Teo S., Lam D.H., Jeyaseelan K., Wang S. (2012). MicroRNA-10b pleiotropically regulates invasion, angiogenicity and apoptosis of tumor cells resembling mesenchymal subtype of glioblastoma multiforme. Cell Death Dis..

[68] Liao H., Bai Y., Qiu S., Zheng L., Huang L., Liu T., Wang X., Liu Y., Xu N., Yan X. (2015). MiR-203 downregulation is responsible for chemoresistance in human glioblastoma by promoting epithelial-mesenchymal transition via SNAI2. Oncotarget.

[69] Xu W., Hu G.Q., Da Costa C., Tang J.H., Li Q.R., Du L., Pan Y.W., Lv S.Q. (2019). Long noncoding RNA UBE2R2-AS1 promotes glioma cell apoptosis via targeting the miR-877-3p/TLR4 axis. Onco Targets Ther..

[70] Blachere N.E., Li Z., Chandawarkar R.Y., Suto R., Jaikaria N.S., Basu S., Udono H., Srivastava P.K. (1997). Heat shock protein-peptide complexes, reconstituted in vitro, elicit peptide-specific cytotoxic T lymphocyte response and tumor immunity. J. Exp. Med..

[71] Binder R.J., Srivastava P.K. (2004). Essential role of CD91 in re-presentation of gp96-chaperoned peptides. Proc. Natl. Acad. Sci. USA.

[72] Takizawa H., Fritsch K., Kovtonyuk L.V., Saito Y., Yakkala C., Jacobs K., Ahuja A.K., Lopes M., Hausmann A., Hardt W.-D. (2017). Pathogen-induced TLR4-TRIF innate immune signaling in hematopoietic stem cells promotes proliferation but reduces competitive fitness. Cell Stem Cell.

[73] Jouhi L., Renkonen S., Atula T., Mäkitie A., Haglund C., Hagström J. (2014). Different toll-like receptor expression patterns in progression toward cancer. Front. Immunol.

[74] Gregg K.A., Harberts E., Gardner F.M., Pelletier M.R., Cayatte C., Yu L., McCarthy M.P., Marshall J.D., Ernst R.K. (2017). Rationally designed TLR4 ligands for vaccine adjuvant discovery. MBio.

[75] Shi M., Chen X., Ye K., Yao Y., Li Y. (2016). Application potential of toll-like receptors in cancer immunotherapy: Systematic review. Medicine.

[76] Apetoh L., Ghiringhelli F., Tesniere A., Obeid M., Ortiz C., Criollo A., Mignot G., Maiuri M.C., Ullrich E., Saulnier P. (2007). Toll-like receptor 4-dependent contribution of the immune system to anticancer chemotherapy and radiotherapy. Nat. Med..

[77] Terashita T., Saito S., Nabeka H., Hato N., Wakisaka H., Shimokawa T., Kobayashi N., Gyo K., Matsuda S. (2013). Prosaposin-derived peptide alleviates ischaemia-induced hearing loss. Acta Otolaryngol..

[78] Kuzmich N.N., Sivak K.V., Chubarev V.N., Porozov Y.B., Savateeva-Lyubimova T.N., Peri F. (2017). TLR4 Signaling Pathway Modulators as Potential Therapeutics in Inflammation and Sepsis. Vaccines (Basel).

[79] Ciafre S.A., Galardi S., Mangiola A., Ferracin M., Liu C.-G., Sabatino G., Negrini M., Maira G., Croce C.M., Farace M.G. (2005). Extensive modulation of a set of microRNAs in primary glioblastoma. Biochem. Biophys. Res. Commun..

[80] Shea A., Harish V., Afzal Z., Chijioke J., Kedir H., Dusmatova S., Roy A., Ramalinga M., Harris B., Blancato J. (2016). MicroRNAs in glioblastoma multiforme pathogenesis and therapeutics. Cancer Med..

[81] Møller H.G., Rasmussen A.P., Andersen H.H., Johnsen K.B., Henriksen M., Duroux M. (2013). A systematic review of microRNA in glioblastoma multiforme: Micro-modulators in the mesenchymal mode of migration and invasion. Mol. Neurobiol..

[82] Ahir B.K., Ozer H., Engelhard H.H., Lakka S.S. (2017). MicroRNAs in glioblastoma pathogenesis and therapy: A comprehensive review. Crit. Rev. Oncol. Hematol..

[83] Godlewski J., Nowicki M.O., Bronisz A., Williams S., Otsuki A., Nuovo G., Raychaudhury A., Newton H.B., Chiocca E.A., Lawler S. (2008). Targeting of the Bmi-1 oncogene/stem cell renewal factor by microRNA-128 inhibits glioma proliferation and self-renewal. Cancer Res..

[84] Lawler S., Chiocca E.A. (2009). Emerging functions of microRNAs in glioblastoma. J. Neurooncol..

[85] Silber J., Lim D.A., Petritsch C., Persson A.I., Maunakea A.K., Yu M., Vandenberg S.R., Ginzinger D.G., James C.D., Costello J.F. (2008). miR-124 and miR-137 inhibit proliferation of glioblastoma multiforme cells and induce differentiation of brain tumor stem cells. BMC Med..

[86] Chan J.A., Krichevsky A.M., Kosik K.S. (2005). MicroRNA-21 is an antiapoptotic factor in human glioblastoma cells. Cancer Res..

[87] Yang G., Zhang R., Chen X., Mu Y., Ai J., Shi C., Liu Y., Shi C., Sun L., Rainov N.G. (2011). MiR-106a inhibits glioma cell growth by targeting E2F1 independent of p53 status. J. Mol. Med..

[88] De Smaele E., Ferretti E., Gulino A. (2010). MicroRNAs as biomarkers for CNS cancer and other disorders. Brainres.

[89] Huse J.T., Brennan C., Hambardzumyan D., Wee B., Pena J., Rouhanifard S.H., Sohn-Lee C., Agami R., Tuschl T., Holland E.C. (2009). The PTEN-regulating microRNA miR-26a is amplified in high-grade gliomaand facilitates gliomagenesis in vivo. Genes Dev..

[90] Corsten M.F., Miranda R., Kasmieh R., Krichevsky A.M., Weissleder R., Shah K. (2007). MicroRNA-21 knockdowndisrupts glioma growthin vivoand displays synergistic cytotoxicity with neural precursor cell deliveredS-TRAIL in human gliomas. Cancer Res..

[91] Wang Q., Li P., Li A., Jiang W., Wang H., Wang J., Xie K. (2019). Plasma specifc miRNAs as predictive biomarkersfor diagnosis and prognosis of glioma. J. Exp. Clin. Cancer Res..

[92] Hermansen S.K., Dahlrot R.H., Nielsen B.S., Hansen S., Kristensen B.W. (2013). MiR-21 expression in the tumorcell compartment holds unfavourable prognostic value in gliomas. J. Neurooncol..

[93] Mazurek M., Litak J., Kamieniak P., Osuchowska I., Maciejewski R., Roliński J., Grajkowska W., Grochowski C. (2020). Micro RNA Molecules as Modulators of Treatment Resistance, Immune Checkpoints Controllers and Sensitive Biomarkers in Glioblastoma Multiforme. Int. J. Mol. Sci..

[94] Taganov K.D., Boldin M.P., Chang K.J., Baltimore D. (2006). NF-kappaB-dependent induction of microRNA miR146, an inhibitor targeted to signaling proteins of innate immune responses. Proc. Natl. Acad. Sci. USA.

[95] Hou J., Wang P., Lin L., Liu X., Ma F., An H., Wang Z., Cao X. (2009). MicroRNA-146a feedback inhibits RIG-I-dependent type I IFN production in macrophages by targeting TRAF6, IRAK1, and IRAK2. J. Immunol..

[96] Shao M., Rossi S., Chelladurai B., Shimizu M., Ntukogu O., Ivan M., Calin G.A., Matei D. (2011). PDGF induced microRNA alterations in cancer cells. Nucleic Acids Res..

[97] Luan S., Sun L., Huang F. (2010). MicroRNA-34a: A novel tumor suppressor in p53-mutant glioma cell line U251. Arch. Med. Res..

[98] Xu M., Li D., Yang C., Ji J.S. (2018). MicroRNA-34a Inhibition of the TLR Signaling Pathway Via CXCL10 Suppresses Breast Cancer Cell Invasion and Migration. Cell Physiol. Biochem..

[99] Xiang W., Tian C., Peng S., Zhou L., Pan S., Deng Z. (2017). Let-7i attenuates human brain microvascular endothelial cell damage in oxygen glucose deprivation model by decreasing toll-like receptor 4 expression. Biochem. Biophys. Res. Commun..

[100] Heneka M.T., McManus R.M., Latz E. (2018). Inflammasome signalling in brain function and neurodegenerative disease. Nat. Rev. Neurosci..

[101] Lippai D., Bala S., Csak T., Kurt-Jones E.A., Szabo G. (2013). Chronic alcohol-induced microRNA-155 contributes to neuroinflammation in a TLR4-dependent manner in mice. PLoS ONE.

[102] Mueller M., Zhou J., Yang L., Gao Y., Wu F., Schoeberlein A., Surbek D., Barnea E.R., Paidas M., Huang Y. (2014). PreImplantation factor promotes neuroprotection by targeting microRNA let-7. Proc. Natl. Acad. Sci. USA.

[103] Lu M., Zhang P.J., Li C.H., Lv Z.-M., Zhang W.-W., Jin C.-H. (2015). miRNA-133 augments coelomocyte phagocytosis in bacteria-challenged Apostichopus japonicus via targeting the TLR component of IRAK-1 in vitro and in vivo. Sci. Rep..

[104] Schulte L.N., Westermann A.J., Vogel J. (2013). Differential activation and functional specialization of miR-146 and miR-155 in innate immune sensing. Nucleic Acids Res..

[105] Chen Y., Chen J., Wang H., Shi J., Wu K., Liu S., Liu Y., Wu J. (2013). HCV-induced miR-21 contributes to evasion of host immune system by targeting MyD88 and IRAK1. PLoS Pathog..

[106] Xu Z., Dong D., Chen X., Huang H., Wen S. (2015). MicroRNA-381 negatively regulates TLR4 signaling in A549 cells in response to LPS stimulation. Biomed. Res. Int..

[107] Park H., Huang X., Lu C., Cairo M.S., Zhou X. (2015). MicroRNA-146a and microRNA-146b regulate human dendritic cell apoptosis and cytokine production by targeting TRAF6 and IRAK1 proteins. J. Biol. Chem..

[108] Arenas-Padilla M., Mata-Haro V. (2018). Regulation of TLR signaling pathways by microRNAs: Implications in inflammatory diseases. Centr. Eur. J. Immunol..

[109] Cherradi N., Feige J.-J., Choi S. (2018). Tristetraprolin (ZFP36) and TIS11B (ZFP36-L1). Encyclopedia of Signaling Molecules.

[110] Anwar M.A., Shah M., Kim J., Choi S. (2019). Recent clinical trends in Toll-like receptor targeting therapeutics. Med. Res. Rev..

[111] Mahlokozera T., Vellimana A.K., Li T., Mao D.D., Zohny Z.S., Kim D.H., Tran D.D., Marcus D.S., Fouke S.J., Campian J.L. (2018). Biological and therapeutic implications of multisector sequencing in newly diagnosed glioblastomas. Neuro-Oncol..

[112] Liu F., Huang J., Liu X., Cheng Q., Luo C., Liu Z. (2020). CTLA-4 correlates with immune and clinical characteristics of glioma. Cancer Cell Int..

[113] Lim M., Xia Y., Bettegowda C., Weller M. (2018). Current state of immunotherapy for glioblastoma. Nat. Rev. Clin. Oncol..

[114] Aslan K., Turco V., Blobner J., Sonner J.K., Liuzzi A.R., Núñez N.G., De Feo D., Kickingereder P., Fischer M., Green E. (2020). Heterogeneity of response to immune checkpoint blockade in hypermutated experimental gliomas. Nat. Commun..

[115] Dong H., Zhu G., Tamada K., Chen L. (1999). B7-H1, a third member of the B7 family, costimulates T-cell proliferation and interleukin-10 secretion. Nat. Med..

[116] Chen J., Jiang C.C., Jin L., Zhang X.D. (2016). Regulation of PD-L1: A novel role of pro-survival signalling in cancer. Ann. Oncol..

[117] Wang L.L., Li Z.H., Hu X.H., Muyayalo K.P., Zhang Y.H., Liao A.H. (2017). The roles of the PD-1/PD-L1 pathway at immunologically privileged sites. Am. J. Reprod. Immunol..

[118] Sims J.S., Ung T.H., Neira J.A., Canoll P., Bruce J.N. (2015). Biomarkers for glioma immunotherapy: The next generation. J. Neurooncol..

[119] Litak J., Mazurek M., Grochowski C., Kamieniak P., Roliński J. (2019). PD-L1/PD-1 Axis in Glioblastoma Multiforme. Int. J. Mol. Sci..

[120] Van Gool S.W. (2015). Brain Tumor Immunotherapy: What have We Learned so Far?. Front. Oncol..

[121] Pfannenstiel L.W., McNeilly C., Xiang C., Kang K., Diaz-Montero C.M., Yu J.S., Gastman B.R. (2018). Combination PD-1 blockade and irradiation of brain metastasis induces an effective abscopal effect in melanoma. Oncoimmunology.

[122] Zhang X., Zhu S., Li T., Liu Y.J., Chen W., Chen J. (2017). Targeting immune checkpoints in malignant glioma. Oncotarget.

[123] Röver L.K., Gevensleben H., Dietrich J., Bootz F., Landsberg J., Goltz D., Dietrich D. (2018). PD-1 (PDCD1) Promoter Methylation Is a Prognostic Factor in Patients With Diffuse Lower-Grade Gliomas Harboring Isocitrate Dehydrogenase (IDH) Mutations. EBioMedicine.

[124] Qian J., Wang C., Wang B., Yang J., Wang Y., Luo F., Xu J., Zhao C., Liu R., Chu Y. (2018). The IFN-γ/PD-L1 axis between T cells and tumor microenvironment: Hints for glioma anti-PD-1/PD-L1 therapy. J. Neuroinflammation.

[125] Goods B.A., Hernandez A.L., Lowther D.E., Lucca L.E., Lerner B.A., Gunel M., Raddassi K., Coric V., Hafler D.A., Love J.C. (2017). Functional differences between PD-1+ and PD-1-CD4+ effector T cells in healthy donors and patients with glioblastoma multiforme. PLoS ONE.

[126] Chinai J.M., Janakiram M., Chen F., Chen W., Kaplan M., Zang X. (2015). New immunotherapies targeting the PD-1 pathway. Trends Pharm. Sci..

[127] Lenting K., Verhaak R., Ter Laan M., Wesseling P., Leenders W. (2017). Glioma: Experimental models and reality. Acta Neuropathol..

[128] Beswick E.J., Johnson J.R., Saada J.I., Humen M., House J., Dann S., Qiu S., Brasier A.R., Powell D.W., Reyes V.E. (2014). TLR4 activation enhances the PD-L1-mediated tolerogenic capacity of colonic CD90+ stromal cells. J. Immunol..

[129] Wolfle S.J., Strebovsky J., Bartz H., Sahr A., Arnold C., Kaiser C., Dalpke A.H., Heeg K. (2011). PD-L1 expression on tolerogenic APCs is controlled by STAT-3. Eur. J. Immunol..

[130] Zhao S., Sun M., Meng H., Ji H., Liu Y., Zhang M., Li H., Li P., Zhang Y., Zhang Q. (2019). TLR4 expression correlated with PD-L1 expression indicates a poor prognosis in patients with peripheral T-cell lymphomas. Cancer Manag. Res..

[131] He X., Semenov M., Tamai K., Zeng X. (2004). LDL receptor related proteins 5 and 6 in Wnt/β-catenin signaling: Arrows point the way. Development.

[132] Minami Y., Oishi I., Endo M., Nishita M. (2010). Ror-family receptor tyrosine kinases in noncanonical Wnt signaling: Their implications in developmental morphogenesis and human diseases. Dev. Dyn..

[133] Peradziryi H., Tolwinski N.S., Borchers A. (2012). The many roles of PTK7: A versatile regulator of cell–cell communication. Arch. Biochem. Biophys..

[134] Fradkin L.G., Dura J.M., Noordermeer J.N. (2010). Ryks: New partners for Wnts in the developing and regenerating nervous system. Trends Neurosci..

[135] Jing L., Lefebvre J.L., Gordon L.R., Granato M. (2009). Wnt signals organize synaptic prepattern and axon guidance through the zebrafish unplugged/MuSK receptor. Neuron.

[136] Kikuchi A., Yamamoto H., Sato A., Matsumoto S. (2011). Discovery of MUSK as a new WNT (co-?) receptor in axon guidance. New insights into the mechanism of Wnt signaling pathway activation. Int. Rev. Cell Mol. Biol..

[137] Cruciat C.M., Niehrs C. (2013). Secreted and transmembrane Wnt inhibitors and activators. Cold Spring Harb. Perspect. Biol..

[138] Abbott N.J., Patabendige A.A., Dolman D.E., Yusof S.R., Begley D.J. (2010). Structure and function of the blood–brain barrier. Neurobiol. Dis..

[139] Butt A.M., Jones H.C., Abbott N.J. (1990). Electrical resistance across the blood–brain barrier in anaesthetized rats: A developmental study. J. Physiol..

[140] Wolburg H., Lippoldt A. (2002). Tight junctions of the blood–brain barrier: Development, composition and regulation. Vasc. Pharmacol..

[141] Bauer H.C., Bauer H., Lametschwandtner A., Amberger A., Ruiz P., Steiner M. (1993). Neovascularization and the appearance of morphological characteristics of the blood–brain barrier in the embryonic mouse central nervous system. Brain Res. Dev. Brain Res..

[142] Amasheh S., Schmidt T., Mahn M., Florian P., Mankertz J., Tavalali S., Gitter A.H., Schulzke J.D., Fromm M. (2005). Contribution of claudin-5 to barrier properties in tight junctions of epithelial cells. Cell Tissue Res..

[143] Ohtsuki S., Sato S., Yamaguchi H., Kamoi M., Asashima T., Terasaki T. (2007). Exogenous expression of claudin-5 induces barrier properties in cultured rat brain capillary endothelial cells. J. Cell Physiol..

[144] Nitta T., Hata M., Gotoh S., Seo Y., Sasaki H., Hashimoto N., Furuse M., Tsukita S. (2003). Size-selective loosening of the blood–brain barrier in claudin-5-deficient mice. J. Cell Biol..

[145] Zuccarini M., Giuliani P., Ziberi S., Carluccio M., Iorio P.D., Caciagli F., Ciccarelli R. (2018). The Role of Wnt Signal in Glioblastoma Development and Progression: A Possible New Pharmacological Target for the Therapy of This Tumor. Genes (Basel).

[146] Rajakulendran N., Rowland K.J., Selvadurai H.J., Ahmadi M., Park N.I., Naumenko S., Dolma S., Ward R.J., So M., Lee L. (2019). Wnt and Notch signaling govern self-renewal and differentiation in a subset of human glioblastoma stem cells. Genes Dev..

[147] De Robertis A., Valensin S., Rossi M., Tunici P., Verani M., De Rosa A., Giordano C., Varrone M., Nencini A., Pratelli C. (2013). Identification and characterization of a small-molecule inhibitor of Wnt signaling in glioblastoma cells. Mol. Cancer Ther..

[148] Broekman M.L., Maas S.L.N., Abels E.R., Mempel T.R., Krichevsky A.M., Breakefield X.O. (2018). Multidimensional communication in the microenvirons of glioblastoma. Nat. Rev. Neurol..

[149] Zarnescu O., Brehar F.M., Chivu M., Ciurea A.V. (2008). Immunohistochemical localization of caspase-3, caspase-9 and Bax in U87 glioblastoma xenografts. J. Mol. Hist..

[150] Tirapelli L.F., Bolini P.H., Tirapelli D.P., Peria F.M., Becker A.N., Saggioro F.P., Carlotti C.G. (2010). Caspase-3 and Bcl-2 expression in glioblastoma: An immunohistochemical study. Arq. Neuropsiquiatr..

[151] Gdynia G., Grund K., Eckert A., Böck B.C., Funke B., Macher- Goeppinger S., Sieber S., Herold-Mende C., Wiestler B., Wiestler O.D. (2007). Basal caspase activity promotes migration and invasiveness in glioblastoma cells. Mol. Cancer Res..

[152] Jia W., Lu R., Martin T.A., Jiang W.G. (2014). The role of claudin-5 in blood-brain barrier (BBB) and brain metastases (review). Mol. Med. Rep..

[153] Hara K., Kageji T., Mizobuchi Y., Kitazato K.T., Okazaki T., Fujihara T., Nakajima K., Mure H., Kuwayama K., Hara T. (2015). Blocking of the interaction between Wnt proteins and their co-receptors contributes to the anti-tumor effects of adenovirus-mediated DKK3 in glioblastoma. Cancer Lett..

[154] Che F., Yin J., Quan Y., Xie X., Heng X., Du Y., Wang L. (2017). TLR4 interaction with LPS in glioma CD133+ cancer stem cells induces cell proliferation, resistance to chemotherapy and evasion from cytotoxic T lymphocyte-induced cytolysis. Oncotarget.

[155] Rajesh Y., Pal I., Banik P., Chakraborty S., Borkar S.A., Dey G., Mukherjee A., Mandal M. (2017). Insights into molecular therapy of glioma: Current challenges and next generation blueprint. Acta Pharm. Sin..

[156] Dzaye O., Hu F., Derkow K., Haage V., Euskirchen P., Harms C., Lehnardt S., Synowitz M., Wolf S.A., Kettenmann H. (2016). Glioma Stem Cells but Not Bulk Glioma Cells Upregulate IL-6 Secretion in Microglia/Brain Macrophages via Toll-like Receptor 4 Signaling. J. Neuropathol. Exp. Neurol..

[157] Ma Q., Long W., Xing C., Chu J., Luo M., Wang H.Y., Liu Q., Wang R.F. (2018). Cancer Stem Cells and Immunosuppressive Microenvironment in Glioma. Front. Immunol..

[158] Testa U., Castelli G., Pelosi E. (2018). Genetic Abnormalities, Clonal Evolution, and Cancer Stem Cells of Brain Tumors. Med. Sci. (Basel).

[159] Widera D., Martínez Aguilar R., Cottrell G.S. (2019). Toll-like receptor 4 and protease-activated receptor 2 in physiology and pathophysiology of the nervous system: More than just receptor cooperation?. Neural Regen. Res..

[160] Alvarado A.G., Thiagarajan P.S., Mulkearns-Hubert E.E., Silver D.J., Hale J.S., Alban T.J., Turaga S.M., Jarrar A., Reizes O., Longworth M.S. (2017). Glioblastoma Cancer Stem Cells Evade Innate Immune Suppression of Self-Renewal through Reduced TLR4 Expression. Cell Stem Cell.

[161] Zhou H., Bao J., Zhu X., Dai G., Jiang X., Jiao X., Sheng H., Huang J., Yu H. (2018). Retinoblastoma Binding Protein 5 Correlates with the Progression in Hepatocellular Carcinoma. Biomed Res. Int..

[162] Ghasemzadeh A., Bivalacqua T.J., Hahn N.M., Drake C.G. (2016). New Strategies in Bladder Cancer: A Second Coming for Immunotherapy. Clin. Cancer Res..

[163] Ou T., Lilly M., Jiang W. (2018). The Pathologic Role of Toll-Like Receptor 4 in Prostate Cancer. Front. Immunol..

[164] Li X.X., Sun G.P., Meng J., Li X., Tang Y.X., Li Z., Wang M.F., Liang G.F., Lu X.B. (2014). Role of toll-like receptor 4 in colorectal carcinogenesis: A meta-analysis. PLoS ONE.

[165] Dapito D.H., Mencin A., Gwak G.Y., Pradere J.P., Jang M.K., Mederacke I., Caviglia J.M., Khiabanian H., Adeyemi A., Bataller R. (2012). Promotion of hepatocellular carcinoma by the intestinal microbiota and TLR4. Cancer Cell.

[166] Thuringer D., Hammann A., Benikhlef N., Fourmaux E., Bouchot A., Wettstein G., Solary E., Garrido C. (2011). Transactivation of the epidermal growth factor receptor by heat shock protein 90 via Toll-like receptor 4 contributes to the migration of glioblastoma cells. J. Biol. Chem..

[167] Chicoine M.R., Zahner M., Won E.K., Kalra R.R., Kitamura T., Perry A., Higashikubo R. (2007). The in vivo antitumoral effects of lipopolysaccharide against glioblastoma multiforme are mediated in part by Toll-like receptor 4. Neurosurgery.

[168] Kawanishi Y., Tominaga A., Okuyama H., Fukuoka S., Taguchi T., Kusumoto Y., Yawata T., Fujimoto Y., Ono S., Shimizu K. (2013). Regulatory effects of Spirulina complex polysaccharides on growth of murine RSV-M glioma cells through Toll-like receptor 4. Microbiol. Immunol..

[169] Shinohara H., Yagita H., Ikawa Y., Oyaizu N. (2000). Fas drives cell cycle progression in glioma cells via extracellular signal-regulated kinase activation. Cancer Res..

[170] Wu Y., Sun L., Zou W., Xu J., Liu H., Wang W., Yun X., Gu J. (2012). Prosaposin, a regulator of estrogen receptor alpha, promotes breast cancer growth. Cancer Sci..

[171] Jiang Y., Zhou J., Luo P., Gao H., Ma Y., Chen Y.S., Li L., Zou D., Zhang Y., Jing Z. (2018). Prosaposin promotes the proliferation and tumorigenesis of glioma through toll-like receptor 4 (TLR4)-mediated NF-κB signaling pathway. EBioMedicine.

[172] Bowles A.P., Perkins E. (1999). Long-term remission of malignant brain tumors after intracranial infection: A report of four cases. Neurosurgery.

[173] De Bonis P., Albanese A., Lofrese G., de Waure C., Mangiola A., Pettorini B.L., Pompucci A., Balducci M., Fiorentino A., Lauriola L. (2011). Postoperative infection may influence survival in patients with glioblastoma: Simply a myth. Neurosurgery.

[174] Bohman L.E., Gallardo J., Hankinson T.C., Waziri A.E., Mandigo C.E., McKhann G.M., Sisti M.B., Canoll P., Bruce J.N. (2009). The survival impact of postoperative infection in patients with glioblastoma multiforme. Neurosurgery.

[175] Agrawal N., Bettegowda C., Cheong I., Geschwind J.F., Drake C.G., Hipkiss E.L., Tatsumi M., Dang L.H., Diaz L.A., Pomper M. (2004). Bacteriolytic therapy can generate a potent immune response against experimental tumors. Proc. Natl. Acad. Sci. USA.

[176] Wang H., Cho C.H. (2010). Effect of nf-kappab signaling on apoptosis in chronic inflammation-associated carcinogenesis. Curr. Cancer Drug Targets.

[177] Baldwin A.S. (2012). Regulation of cell death and autophagy by ikk and nf-kappab: Critical mechanisms in immune function and cancer. Immunol. Rev..

[178] Guo S., Nighot M., Al-Sadi R., Alhmoud T., Nighot P., Ma T.Y. (2015). Lipopolysaccharide Regulation of Intestinal Tight Junction Permeability Is Mediated by TLR4 Signal Transduction Pathway Activation of FAK and MyD88. J. Immunol..

[179] Kondo Y., Ikeda K., Tokuda N., Nishitani C., Ohto U., Akashi-Takamura S., Ito Y., Uchikawa M., Kuroki Y., Taguchi R. (2013). TLR4-MD-2 complex is negatively regulated by an endogenous ligand, globotetraosylceramide. Proc. Natl. Acad. Sci. USA.

[180] Wang Y., Su L., Morin M.D., Jones B.T., Whitby L.R., Surakattula M.M., Huang H., Shi H., Choi J.H., Wang K.W. (2016). TLR4/MD-2 activation by a synthetic agonist with no similarity to LPS. Proc. Natl. Acad. Sci. USA.

[181] Matzner P., Sorski L., Shaashua L., Elbaz E., Lavon H., Melamed R., Rosenne E., Gotlieb N., Benbenishty A., Reed S.G. (2016). Perioperative treatment with the new synthetic TLR-4 agonist GLA-SE reduces cancer metastasis without adverse effects. Int. J. Cancer.

[182] Coler R.N., Day T.A., Ellis R., Piazza F.M., Beckmann A.M., Vergara J., Rolf T., Lu L., Alter G., Hokey D. (2018). TBVPX-113 Study Team. The TLR-4 agonist adjuvant, GLA-SE, improves magnitude and quality of immune responses elicited by the ID93 tuberculosis vaccine: First-in-human trial. NPJ Vaccines.

[183] Fensterheim B.A., Young J.D., Luan L., Kleinbard R.R., Stothers C.L., Patil N.K., McAtee-Pereira A.G., Guo Y., Trenary I., Hernandez A. (2018). The TLR4 Agonist Monophosphoryl Lipid A Drives Broad Resistance to Infection via Dynamic Reprogramming of Macrophage Metabolism. J. Immunol..

[184] Stark R., Choi H., Koch S., Lamb F., Sherwood E. (2015). Monophosphoryl lipid A inhibits the cytokine response of endothelial cells challenged with LPS. Innate Immun..

[185] Gao J., Guo Z. (2018). Progress in the synthesis and biological evaluation of lipid A and its derivatives. Med. Res. Rev..

[186] Carpentier A., Laigle-Donadey F., Zohar S., Capelle L., Behin A., Tibi A., Martin-Duverneuil N., Sanson M., Lacomblez L., Taillibert S. (2006). Phase 1 trial of a CpG oligodeoxynucleotide for patients with recurrent glioblastoma. Neuro Oncol..

[187] Zhou M., McFarland-Mancini M.M., Funk H.M., Husseinzadeh N., Mounajjed T., Drew A.F. (2009). Toll-like receptor expression in normal ovary and ovarian tumors. Cancer Immunol. Immunother..

[188] Andreani V., Gatti G., Simonella L., Rivero V., Maccioni M. (2007). Activation of Toll-like receptor 4 on tumor cells in vitro inhibits subsequent tumor growth in vivo. Cancer Res..

[189] Alibashe-Ahmed M., Roger T., Serre-Beinier V., Berishvili E., Reith W., Bosco D., Berney T. (2019). Macrophage migration inhibitory factor regulates TLR4 expression and modulates TCR/CD3-mediated activation in CD4+ T lymphocytes. Sci. Rep..

[190] Kumar R., de Mooij T., Peterson T.E., Kaptzan T., Johnson A.J., Daniels D.J., Parney I.F. (2017). Modulating glioma-mediated myeloid-derived suppressor cell development with sulforaphane. PLoS ONE.

[191] Li W., Holsinger R.M., Kruse C.A., Flügel A., Graeber M.B. (2013). The potential for genetically altered microglia to influence glioma treatment. CNS Neurol. Disord. Drug Targets.

[192] Roger T., David J., Glauser M.P., Calandra T. (2001). MIF regulates innate immune responses through modulation of Toll-like receptor 4. Nature.

[193] Ohta S., Yaguchi T., Okuno H., Chneiweiss H., Kawakami Y., Okano H. (2016). CHD7 promotes proliferation of neural stem cells mediated by MIF. Mol. Brain.

[194] Roger T., Schneider A., Weier M., Sweep F.C., Le Roy D., Bernhagen J., Calandra T., Giannoni E. (2016). High expression levels of macrophage migration inhibitory factor sustain the innate immune responses of neonates. Proc. Natl. Acad. Sci. USA.

[195] Ohkawara T., Takeda H., Nishihira J., Miyashita K., Nihiwaki M., Ishiguro Y., Takeda K., Akira S., Iwanaga T., Sugiyama T. (2005). Macrophage migration inhibitory factor contributes to the development of acute dextran sulphate sodium-induced colitis in Toll-like receptor 4 knockout mice. Clin. Exp. Immunol..

[196] Schwenkgrub J., Zaremba M., Mirowska-Guzel D., Kurkowska-Jastrzebska I. (2017). Ibudilast: A nonselective phosphodiesterase inhibitor in brain disorders. Postepy Hig. Med. Dosw..

[197] Ha W., Sevim-Nalkiran H., Zaman A.M., Matsuda K., Khasraw M., Nowak A.K., Chung L., Baxter R.C., McDonald K.L. (2019). Ibudilast sensitizes glioblastoma to temozolomide by targeting Macrophage Migration Inhibitory Factor (MIF). Sci. Rep..

[198] Roger T., Froidevaux C., Martin C., Calandra T. (2003). Macrophage migration inhibitory factor (MIF) regulates host responses to endotoxin through modulation of Toll-like receptor 4 (TLR4). J. Endotoxin Res..

[199] Basu S., Binder R.J., Ramalingam T., Srivastava P.K. (2001). CD91 is a common receptor for heat shock proteins gp96, hsp90, hsp70, and calreticulin. Immunity.

[200] Castellino F., Boucher P.E., Eichelberg K., Mayhew M., Rothman J.E., Houghton A.N., Germain R.N. (2000). Receptor-mediated uptake of antigen/heat shock protein complexes results in major histocompatibility complex class I antigen presentation via two distinct processing pathways. J. Exp. Med..

[201] Matsutake T., Sawamura T., Srivastava P.K. (2010). High efficiency CD91- and LOX-1-mediated representation of gp96-chaperoned peptides by MHC II molecules. Cancer Immun..

[202] Berwin B., Hart J.P., Pizzo S.V., Nicchitta C.V. (2002). Cutting edge: CD91-independent cross-presentation of GRP94(gp96)-associated peptides. J. Immunol..

[203] Panjwani N.N., Popova L., Srivastava P.K. (2002). Heat shock proteins gp96 and hsp70 activate the release of nitric oxide by APCs. J. Immunol..

[204] Srivastava P. (2002). Roles of heat-shock proteins in innate and adaptive immunity. Nat. Rev. Immunol..

[205] Crane C.A., Han S.J., Ahn B., Oehlke J., Kivett V., Fedoroff A., Butowski N., Chang S.M., Clarke J., Berger M.S. (2013). Individual patient-specific immunity against high-grade glioma after vaccination with autologous tumor derived peptides bound to the 96 KD chaperone protein. Clin. Cancer Res..

[206] Bloch O., Crane C.A., Fuks Y., Kaur R., Aghi M.K., Berger M.S., Butowski N.A., Chang S.M., Clarke J.L., McDermott M.W. (2014). Heat-shock protein peptide complex-96 vaccination for recurrent glioblastoma: A phase II, single-arm trial. Neuro-Oncology.

[207] Ampie L., Choy W., Lamano J.B., Fakurnejad S., Bloch O., Parsa A.T. (2015). Heat shock protein vaccines against glioblastoma: From bench to bedside. J. Neurooncol..

[208] Khansur E., Shah A.H., Lacy K., Komotar R.J. (2019). Novel Immunotherapeutics for Treatment of Glioblastoma: The Last Decade of Research. Cancer Investig..

